# Can Optic Flow Further Stimulate Treadmill-Elicited Stepping in Newborns?

**DOI:** 10.3389/fpsyg.2021.665306

**Published:** 2021-05-13

**Authors:** Marianne Barbu-Roth, Kim Siekerman, David I. Anderson, Alan Donnelly, Viviane Huet, François Goffinet, Caroline Teulier

**Affiliations:** ^1^Integrative Neuroscience and Cognition Center, Université de Paris – CNRS, Paris, France; ^2^Department of Physical Education and Sport Sciences, University of Limerick, Limerick, Ireland; ^3^Marian Wright Edelman Institute, San Francisco State University, San Francisco, CA, United States; ^4^Université de Paris, AP-HP, Paris, France; ^5^CIAMS, Université Paris-Sud, Université Paris-Saclay, Orsay, France; ^6^CIAMS, Université d’Orléans, Orléans, France

**Keywords:** infant, locomotion, motor, neonate, walking, vision

## Abstract

Typically developing 3-day-old newborns take significantly more forward steps on a moving treadmill belt than on a static belt. The current experiment examined whether projecting optic flows that specified forward motion onto the moving treadmill surface (black dots moving on the white treadmill surface) would further enhance forward stepping. Twenty newborns were supported on a moving treadmill without optic flow (No OF), with optic flow matching the treadmill’s direction and speed (Congruent), with optic flow in the same direction but at a faster speed (Faster), and in a control condition with an incoherent optic flow moving at the same speed as in the Congruent condition but in random directions (Random). The results revealed no significant differences in the number or coordination of forward treadmill steps taken in each condition. However, the Faster condition generated significantly fewer leg pumping movements than the Random control condition. When highly aroused, newborns made significantly fewer single steps and significantly more parallel steps and pumping movements. We speculate the null findings may be a function of the high friction material that covered the treadmill surface.

## Introduction

Treadmill training is an effective way to promote the acquisition of independent walking in infants with disabilities. Following Thelen and colleagues’ pioneering work (e.g., Thelen, 1986; Thelen and Ulrich, 1991), several authors have shown that treadmill training not only hastens the onset of independent walking in infants with motor delays, but it also improves step coordination (Ulrich et al., 2001, 2008; Bodkin et al., 2003; Wu et al., 2007; see Teulier et al., 2014 for a review). These findings have raised questions about the optimal time to initiate interventions to maximize their effectiveness, particularly in light of a prevailing consensus that interventions should be initiated as early as possible to take advantage of the window of maximum neural plasticity underlying cerebral development (Cioni et al., 2011; Ismail et al., 2017) and specific behaviors like stepping (Ulrich, 2010; Teulier et al., 2015).

A study by Siekerman et al. (2015) suggests that treadmill-based locomotor interventions for infants at risk of developmental delay could start at birth. The study showed that typically developing 3-day-old infants made significantly more forward steps on a moving treadmill belt than on a static belt. Moreover, the newborns showed a degree of specificity in their response to the speed of the treadmill belt, as they made significantly more steps when the belt moved at 17.2 cm/sec compared to 23.4 cm/sec. Arousal also had an influence on stepping. The steps were more clearly differentiated and coordinated (more alternated or serial) when infants were crying. Knowing that treadmill interventions could potentially be started at birth, an obvious question arises about the optimal conditions to promote early stepping. Treadmill speed and the infant’s arousal level influence the amount and coordination of stepping, but what other factors might enhance stepping?

One factor that might enhance treadmill stepping in the newborn period is the availability of optic flow that specifies how the infant is moving relative to the support surface. Barbu-Roth and colleagues have shown that 3-day-olds will make significantly more steps in the air when held upright above an *optical treadmill* – a checkerboard pattern of optic flow that moves beneath the feet and specifies backward or forward displacement – than when held above a rotating pin-wheel or a static checkerboard pattern (Barbu-Roth et al., 2009, 2014). Moreover, Forma and colleagues have found a similar effect of optic flow on newborn crawling (Forma et al., 2018; see Forma et al., 2019 for a discussion of how stepping and crawling are related). Newborns significantly increased their leg flexion and extension movements when exposed to a checkerboard optic flow pattern back-projected onto the surface underneath the transparent, water-filled pediatric mattress onto which they were placed. In addition, typically developing infants between two and 10 months of age increased their stepping frequency when the treadmill belt on which they were stepping was covered with a black-and-white checkerboard pattern, generating congruent optic flow, compared to when the belt was black or white, minimizing optic flow (Moerchen and Saeed, 2012). A similar finding has been reported for seven to 10-month-old infants with myelomeningocele (Pantall et al., 2011, 2012). Together, these findings suggest that it may be possible to enhance newborn treadmill stepping by exposing infants to optic flows that specify forward translation while they are on a treadmill belt that moves in the same direction. The current experiment sought to test this notion.

The experiment was an extension of the experiment reported by Siekerman et al. (2015). Newborns were supported on a moving treadmill without additional optic flow, or with optic flows (black dots moving on the white treadmill surface) that moved in the same direction and at the same speed as the treadmill belt or at a faster speed, or with an incoherent optic flow with dots that moved at the same speed as the treadmill belt but in random directions. We predicted that step frequency and coordination would be enhanced by the optic flows that moved in the same direction as the treadmill belt, but not by the random optic flow. If visual input exerted a more powerful influence on stepping than the tactile/mechanical input from the treadmill belt, we expected to see higher step frequency and coordination in the faster optic flow condition than in the one that moved at the same speed as the treadmill belt. Finally, consistent with the findings reported by Siekerman et al. (2015), we expected that step frequency and the number of alternated and serial steps would be higher when newborns were crying.

## Materials and Methods

### Participants

Twenty-four newborns were recruited from the maternity ward of the Maternity Port Royal Hospital in Paris. Only healthy infants born with an uncomplicated delivery, a minimum term of 38 weeks, an Apgar score of 10 at the fifth minute and a minimum birth weight of 2,500 grams were selected. Four newborns were excluded from the final sample because they fell asleep during the experiment. The final sample included 20 newborns (12 males), a mean birth weight of 3,367 (±482.8) grams, a mean age of 2.7 (±0.7) days, and a mean term of 40.1 (±0.9) weeks. Ethical approval was obtained from the ethics medical board of Paris-Ile de France and parents and caregivers gave informed consent for infant participation.

### Experimental Set-Up and Materials

**FIGURE 1:**
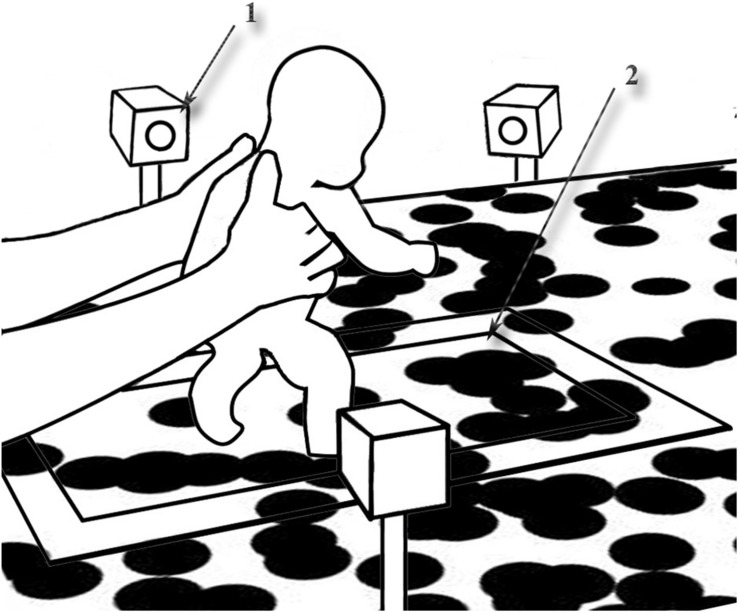
Experimental set-up. (1) camera, (2) treadmill.

The experimental set-up is depicted in Figure 1. An infant sized treadmill (belt surface 0.31 m × 0.59 m) was recessed into a 1 m × 1 m × 2 m (h × w × l) table so that both surfaces were level. The treadmill belt was covered with a white Dycem film (Dycem Ltd., Bristol, United Kingdom) to match the color of the table surface so that the surface appeared to be continuous and to allow the projection of black dots used in the optic flow conditions. Optic flow was created with a MATLAB programme, run on a desk top computer and projected distortion-free onto the table and treadmill surfaces with a Sanyo PLC-XL51 projector (Sanyo Electric Co., Ltd., Osaka, Japan). The visual stimuli consisted of black, 4.5 cm diameter dots on a white surface in the experimental conditions (combined luminance 36.78 cd/m^2^) and a neutral, static gray for the no optic flow (No OF) condition (luminance 14.97 cd/m^2^). There were approximately 1.96 dots randomly positioned per 10 squared cm, with occasional random overlap of the dots leading to the formation of random aggregates ranging from 2 to 10 dots. The dots had a maximal limited lifetime of 16.7 s.

We used the dots stimulus for two reasons. First, the random position of the dots allows us to rule out that the newborn is simply responding to predictable changes in luminance when a regularly patterned stimulus like a black and white checkerboard moves past the eyes. Second, the dots can be programmed to move together in the same direction (coherently) or in different directions (incoherently) to determine whether the infant responds to the amount of dot motion, which is equivalent in coherent and incoherent conditions, or the direction of dot motion. Only coherent motion specifies translation across a surface and so constitutes a “true” optic flow. We have elicited stepping using the same stimulus in an experiment in which the newborns were supported upright on a stationary surface (Barbu-Roth et al., 2016). Four different visual stimuli were presented together with the treadmill moving at 13.4 cm/sec. For the No OF condition, treadmill stimulation was combined with the gray projection; in the Random condition, the dots moved in random directions at 13.4 cm/sec; in the Congruent condition, the dots moved at the same speed and in the same direction as the treadmill belt to mimic forward motion; and in the Faster condition, the dots moved in the same direction but slightly faster (17.24 cm/sec) than the treadmill belt.

The speed of the treadmill and the speeds of the optic flow were selected based on the previous experiments by Siekerman et al. (2015) and Barbu-Roth et al. (2009, 2014). Siekerman and colleagues reported that newborns took more steps when a treadmill belt moved at 17.2 cm/sec than at 13.4 cm/sec or 23.4 cm/sec. Consequently, we chose a treadmill speed of 13.4 cm/sec in the current study to ensure room for an increase in stepping should optic flow have such an effect on stepping. We chose optic flow velocities of 13.4 and 17.2 cm/sec because these velocities are within the range of large field velocities that are well perceived by newborns (Kremenitzer et al., 1979; Jouen et al., 2000) and also within the range of optical flow velocities that have increased air stepping in newborns in previous studies (Barbu-Roth et al., 2009, 2014). Moreover, one of the optical flow velocities matched the speed of the treadmill belt, creating congruency between the optical and mechanical information specifying forward translation, and the other was faster, allowing us to determine if stepping was driven more by the mechanical or the optical information. Consequently, all of the infants were exposed to two optic flow conditions that specified forward displacement and the no optic flow and the random optic flow control conditions that did not specify displacement in any direction. The random optic flow condition was necessary to control for the potential that the newborns engaged in more stepping in the optic flow conditions because the moving dots were arousing. Each trial was 1 min in duration, with a 1- to 2-min break between trials (depending on the state of the infant), for a total testing time of approximately seven to 10 min for each infant.

Two high definition Sony CHDR-CX160 cameras (Sony Corporation, Tokyo, Japan) were placed at 1.22 m height, to the left and right and 85 cm away from the center of the treadmill, capturing movements at 60 Hz. An additional digital mini DV camera (Sony DCR-HC26) was placed at the front left of the infant to record alertness and gaze direction (30 Hz).

### Procedure

Infants were tested after feeding, when they were alert and not crying. They were dressed in a black sleeveless undergarment and supported under the armpits by the experimenter’s hands (upright and angled slightly forwards, with the head and neck supported by the thumbs and the index fingers so their faces were directed toward the treadmill surface). The infants’ feet were in contact with the treadmill belt. A second experimenter, positioned at the other end of the treadmill, monitored the infant for alertness and gaze direction and controlled the visual stimuli. Alertness and gaze direction were confirmed by reviewing the video recordings from the front left camera. The four different conditions were presented in random order. Infants were repositioned at the front end of the treadmill if they had not responded to the treadmill for more than 10 s, to allow the hip to move through flexion and extension.

If infants showed signs of high distress, e.g., desperate crying without pauses, or if the parents indicated their infant was too stressed to continue, they were removed from the treadmill before the end of the trial. As a result, trial times ranged between 34 and 58 s. Missing trials were not repeated because the newborns were only in an optimal condition for testing for a brief period of time. Note that due to the amount of care needed when testing newborns, complete blinding of the experimenter was impossible.

### Data Reduction

Video data were analyzed frame-by frame in SiliconCoach (SiliconCoach Ltd., Dunedin, New Zealand, version 7-0-2-2). Periods with excessive tester movement (e.g., during repositioning of the infant) or touches by a parent (e.g., to encourage or calm the infant) were excluded from the analysis because they led to movements that were not initiated by the infant.

#### Movement Coding

Movement categories were based on the earlier experiment on neonatal treadmill stepping (Siekerman et al., 2015) with two main movements: Pumps and Steps. Pumps included vertical flexion and extension cycles, regardless of whether the foot was in contact with the belt at the start or end of the movement. The pump was similar to a vertical kick, rather than a step, and did not have the potential to contribute to forward propulsion. Steps displayed locomotive potential, with the foot traveling forward for more than half a foot length during swing. A step cycle was defined as the period of time between the foot lifting off the treadmill surface and landing on the surface again, regardless of which part of the foot last left the treadmill and first landed on the treadmill. Step coordination was determined using the same frame by frame analysis that was used to identify steps and pumps. In the Step Coordination category, unilateral steps from one leg were labeled as Single Steps; simultaneous steps with two legs as Parallel Steps; steps that were followed by a step from the opposite leg within 20–80% of its step cycle as Alternated Steps; and steps that were initiated between 80% of the opposite step cycle and 1 s after the finish of that step cycle as Serial Steps.

#### Arousal Coding

Arousal was coded using a combination of all the video footage (from the left, right, and left-front), depending on the direction of the newborn’s face. The scheme used to code arousal was identical to that used in the previous study (Siekerman et al., 2015) and based on Thelen’s (Thelen et al., 1984) six-point scale adaptation of Prechtl’s behavioral states (Prechtl, 1974). Arousal was sampled at 5-s consecutive intervals, recording: (1) gross body movement, (2) if eyes were open or closed, and (3) level of vocalization. Precise mapping between stepping and arousal made it possible to examine whether the stepping parameters changed between less aroused, non-crying states (state 4) and highly aroused, crying states (state 5 and 6). This was done by assigning a state code to each Step and Pump, based on the arousal level of the 5-s time window in which the majority of the movement occurred. Note, we collapsed crying states (5 and 6) because it was very difficult to distinguish the different levels of intensity of crying.

### Statistical Analysis

Non-parametric tests were used (Friedman tests and Signed-rank Wilcoxon tests for between-subject effects and for the arousal analysis) because most parameters were not normally distributed. The distributions were normal for the analysis of the effects of condition order on arousal level, therefore parametric tests were used for this analysis. If an infant did not present with both crying and non-crying arousal states during testing, they were not included in the arousal analysis. Consequently, the sample sizes for the arousal analysis were smaller than those for the condition analysis. Significance level was set at 0.05. Note that due to its exploratory nature, no *post hoc* corrections for multiple comparisons were made in this study.

### Reliability of Coding

In this study, 20% of the trials were re-coded by the original coder and 20% by a second experienced coder. The original coder had considerable experience, having already coded treadmill stepping in previous studies (*n* = 48 infants in total). First, we verified if each coder observed the same steps. Then, for each step observed by both coders, the observations for each subcategory were compared. The total agreement score was divided by the total number of comparisons, resulting in an inter-rater reliability of 86.1%, and an intra-rater reliability of 87.3%. To examine the inter and intra-coder reliability for arousal, 20% of trials were re-coded by a second experimenter and the entire experiment was recoded by the original coder. The state code assigned to each 5-s window was compared between the two coders or, in case of intra-coder reliability, between first and second coding. The agreement criterion was met if the states were the same. Additionally, because the number of options was the same across the entire study, it was possible to calculate a value for κ. The reliability between coders was good, with a percent agreement of 70.5% (*κ* = 0.601). Intra-rater agreement was higher, with a percent agreement of 87.3% (*κ* = 0.876).

## Results

### Combined Optic Flow and Treadmill Effects on Stepping

**FIGURE 2:**
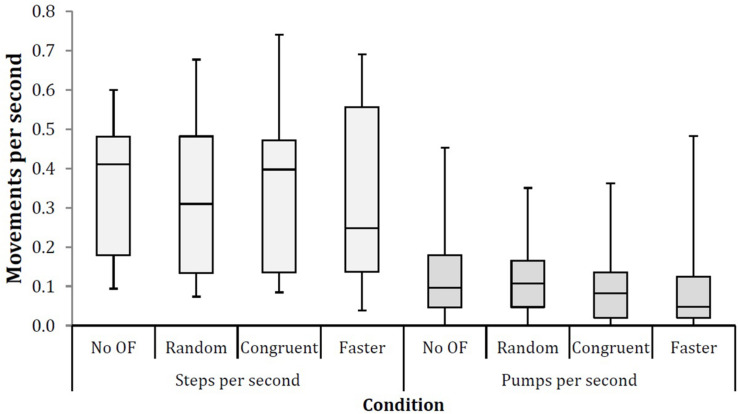
Box plots of step rate (left) and pump rate (right) in the four experimental conditions. Movement rates are expressed per second.

**FIGURE 3:**
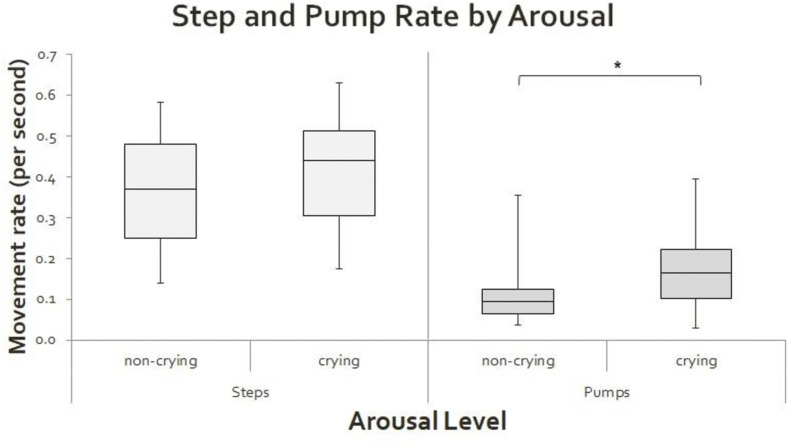
Box plots of step rate (left) and pump rate (right) when infants were crying and not crying. Movement rates are expressed per second. The asterisk (*) indicates a significant difference between comparisons, *p* < 0.05.

The main results are presented in Table 1 and Figures 2,3. The median number of steps per second ranged from 0.248 steps per second in the Faster condition to 0.411 steps per second in the No OF condition (Table 1). A Friedman test showed no significant differences between conditions (χ^2^ = 1.320, *p* = 0.724). However, a significant difference was found for pumps per second: *post hoc* tests (Wilcoxon Signed-rank) showed that more pumps per second were made in the Random condition (median 0.108 pumps per second) than in the Faster condition (median 0.048 pumps per second; χ^2^ = 12.076, *p* = 0.007). No significant differences were observed for the percentage of steps relative to pumps across conditions (χ^2^ = 7.045, *p* = 0.070), though there was a strong trend toward more steps and fewer pumps in the congruent and faster optic flow conditions than in the random and no optic flow control conditions.

**TABLE 1:** Descriptive and Inferential Statistics for Parameters Measured in the No OF, Random, Congruent and Faster Optic Flow Conditions and When Infants Were Crying or Not Crying.

	Parameter	Optic flow condition							Arousal level				
		*χ2*	*n*	*P-value*	*Medians*				*Z-value*	*n*	*P-value*	*Medians*	
					No OF	Random	Congruent	Faster				Not Crying	Crying
Movement type	Step (sec^–1^)	1.32	20	0.724	0.411	0.310	0.397	0.248	−1.161	14	0.245	0.370	0.439
	Pump (sec^–1^)	12.076	20	**0.007**	0.096	0.108	0.082	0.048	−2.229	14	**0.026**	0.093	0.163
	Step (%)	7.045	20	0.070	78.08	73.62	84.52	85.71	−1.852	14	0.064	80.54	71.01
Step Coordination	Single Steps (%)	3.523	20	0.318	55.34	59.85	51.49	53.94	−2.919	14	**0.004**	61.88	39.77
	Parallel Steps (%)	0.397	20	0.941	22.94	22.5	21.11	19.72	−2.261	14	**0.024**	15.67	35.42
	Alternated Steps (%)	1.401	20	0.705	15.23	7.92	3.85	4.84	−1.538	14	0.124	13.58	22.02
	Serial Steps (%)	0.509	20	0.917	0	0	0	0	−1.503	14	0.133	3.73	2.38

**n* refers to the number of infants that contributed data to the parameter. Significant *p-*values are highlighted in bold.*

For Coordination, no significant differences were found between the different conditions. There were no differences for the median percentages of single (χ^2^ = 3.523, *p* = 0.318), parallel (χ^2^ = 0.397, *p* = 0.941), alternated (χ^2^ = 1.401, *p* = 0.705) or serial steps (χ^2^ = 0.509, *p* = 0.917).

### Arousal Effects on Stepping

A repeated measures ANOVA showed no significant differences for mean arousal level between conditions, *F*(3,51) = 0.298, *p* = 0.827.

Newborns did not produce a significantly different median number of steps per second (*Z* = −1.161, *p* = 0.245) when they were crying compared to when they were not crying. However, the median number of pumps per second was higher when newborns cried (0.163 pumps per second) compared to when they were in alert, non-crying states (0.093 pumps per second; *Z* = −2.229, *p* = 0.026) (see Figure 3). No significant step percentage differences were observed between the different arousal levels (*Z* = −1.852, *p* = 0.064), though a strong trend toward more stepping and less pumping was observed in the non-crying states compared to the crying-states.

Step Coordination was affected by arousal. When the newborns were crying relative to not crying, they had a significantly smaller percentage of single steps (39.77% compared to 61.88%, respectively; *Z* = −2.919, *p* = 0.004) but a significantly higher percentage of parallel steps (35.42% compared to 15.67%, respectively; *Z* = −2.261, *p* = 0.024). There were no significant differences for alternated steps (*Z* = −1.538, *p* = 0.124) or serial steps (*Z* = −1.503, *p* = 0.133) as a function of crying.

## Discussion

The absence of improvement in step count or coordination failed to confirm the expectation that adding optic flows that specified forward motion to a moving treadmill would enhance newborn stepping. Similarly, no evidence was found to support the hypothesis that the faster optic flow would enhance stepping relative to the optic flow moving at the same rate as the treadmill belt. The effects of arousal on stepping also failed to conform to expectations. More pumps were seen when newborns were crying, but not more steps. In addition, fewer single steps and more parallel steps were seen when infants were crying but not more alternating steps. Two factors potentially explain why optic flow had no effect on stepping: (1) stepping was driven primarily by the treadmill because the coupling between vision (optic flow) and stepping was not robust enough to exert an additional influence on behavior, and (2) the high friction material on the treadmill belt enhanced the effect of the treadmill belt. We will address each explanation in turn.

### The Rudimentary Coupling Between Vision and Stepping

Despite showing that optic flow enhanced air-stepping in newborns, Barbu-Roth et al. (2009, 2014) have argued that the coupling between vision and stepping is rudimentary, at best. The coupling actually seems to weaken over the first couple of months of life (Barbu-Roth et al., 2015; Anderson et al., 2016), much like the stepping pattern itself when tested on a static surface, only to strengthen as the infant gains experience using vision to control movement. This idea is consistent with prior work showing that optic flow on a treadmill belt is more likely to facilitate stepping in older infants than younger infants (Pantall et al., 2011, 2012; Moerchen and Saeed, 2012).

Improvements in the visual control of locomotion appear to parallel improvements in the visual control of posture (Anderson et al., 2004), both of which are modulated by improvements in the ability to differentiate patterns of optic flow (Gilmore et al., 2004). The visual control of posture and locomotion may take longer to develop than somatosensory-based or vestibular-based control. For example, seated 4-month-old infants displayed poorly organized muscular responses in the neck and trunk when they were rapidly translated forward and backward, but showed much more appropriate responses when they were deprived of vision by opaque goggles (Woollacott et al., 1989). Moreover, notable changes in the infant’s ability to differentiate and utilize different patterns of optic flow for postural control have been documented prior to the onset of independent locomotion (e.g., Shirai and Imura, 2014) and after the onset of independent locomotion (Higgins et al., 1996; Lejeune et al., 2006; Uchiyama et al., 2008; Dahl et al., 2013). Reorganizations in the visual control of posture have also been documented following the acquisition of other notable motor milestones, like sitting and standing (Foster et al., 1996).

A poorly developed ability to integrate multiple sources of information or detect higher-order invariants in multisensory patterns of stimulation may also have compromised the newborns’ ability to utilize optic flow to increase stepping. We expected that the newborns could integrate the tactile input from the treadmill with the visual input from the optic flow to develop a coherent percept of forward motion across the surface. Our expectations may have been inappropriate given that Corbetta and Snapp-Childs (2009) reported that infants as old as 6 to 9 months of age had problems utilizing vision and touch simultaneously to plan hand and finger movements in a grasping task. They claimed that infants “cannot quite parse the sensory information diligently to adjust their response pattern, and therefore continue to respond on the basis of their motor tendencies” (p. 57). If infants older than 6 months had problems using two sources of information simultaneously, then it is highly probable that newborns would have similar, and likely more severe, problems. It is quite possible that the infants in the optic flow conditions experienced a sensory overload that interfered with their ability to make stepping movements on the treadmill. Nevertheless, we cannot completely rule out that any effects we saw in this study were a combination of influences from the mechanical pull of the treadmill and the optic flow and somatosensory stimulation.

Finally, it should be noted that the moving dots that comprised the optic flow stimulus in the present study was a different stimulus from the checkerboard pattern used in Barbu-Roth et al. (2009), where air stepping rates were significantly higher when the checkerboard moved beneath the newborns’ feet than when a black and white pinwheel rotated beneath their feet. Despite the differences, we are confident that the coherently moving dots stimulus was capable of eliciting a stepping response in the newborns because we were able to elicit stepping in response to the same visual stimulus in a group of newborns who were supported in the upright position with their feet in contact with a static surface (Barbu-Roth et al., 2016). Moreover, we also have to consider the failure to replicate the effects of arousal on step rates reported by Siekerman et al. (2015). When highly aroused, the newborns in the Siekerman et al. (2015) study made significantly more stepping movements and their steps were more coordinated. These findings suggest that immaturity in the integration of multiple sources of information might not be the primary reason for the null results in the current study. What other factors might then be at play?

### The High Friction Material on the Treadmill Surface Facilitated Stepping

A speculative explanation for the null effects of optic flow on forward stepping was the unanticipated effect of covering the treadmill belt with Dycem to provide a continuous white surface onto which to project the optic flows. The Dycem may also explain why arousal had no effect on stepping. Dycem is a high friction polymer used in therapeutic settings to reduce slipperiness of a surface. It is important to note that previous experiments have shown that Dycem facilitates the rate of stepping in older infants and increases the duration of the stance phase in the step cycle (Pantall et al., 2011, 2012). Based on these prior findings, we speculate that stepping rates may have approached ceiling levels in the current experiment because the Dycem helped to ensure that stepping was primarily driven by the mechanical pull of the treadmill belt.

A comparison of the step rate reported by Siekerman et al. (2015) when the treadmill belt moved at 13.4 cm/sec (without Dycem) compared to the step rate in the current experiment in the equivalent 13.4 cm/sec condition without optic flow (but with Dycem) provided further support for the idea that the stepping rates in the current experiment were at ceiling because of the Dycem material. The comparison revealed that the step rate was over double in the current experiment compared to the Siekerman et al. experiment (0.41 vs. 0.19 steps per second or 24.6 vs. 11.4 steps per minute). In fact, without Dycem, the step rate obtained by Siekerman et al. on a 13.4 cm/sec moving treadmill (2015) was similar to the rates obtained on a static surface in previous studies on newborns (8 steps/min in Barbu-Roth et al., 2009 and 10 steps/min in Barbu-Roth et al., 2014).

A comparison of the performance of newborns in the current experiment with the maximum stepping observed in previous studies on newborns and older infants provides further evidence that Dycem may have pushed stepping rates toward a ceiling. Interestingly, the maximum number of steps obtained on a treadmill at 1 month of age is 27 steps/min. It increases to 36 steps/min at 3 months and 42 steps/min at 6 months (Teulier et al., 2009). In essence, there is a linear increase in the number of steps on a treadmill without Dycem from birth (11.4 steps/min in Siekerman et al., 2015) to 6 months of age (42 steps/min in Teulier et al., 2009). In contrast, the step rate of 24.6 steps/min obtained with Dycem in our current experiment is a remarkable rate as it is extremely close to the rate at 1 month of age.

Finally, the Dycem might explain the selective nature of the effects of arousal on leg movements. High arousal (crying) had no effect on step counts but significantly increased the number of pumps. Notably, newborns made significantly more forward steps and pumps when they were crying compared to when they were not crying in the Siekerman et al. (2015) experiment. Because the pumps are primarily vertical movements that involve limited contact between the foot and the treadmill surface, this category of leg movement was perhaps the only one that was open to modification by some factor other than the treadmill belt in the current experiment.

In summary, adding optic flow to a moving treadmill belt did not enhance the amount and coordination of newborn forward stepping in the context in which stepping was elicited in the current experiment. The absence of an additive effect of optic flow and treadmill movement may stem from the rudimentary nature of the coupling between vision and stepping or from the newborn’s limited capacity to simultaneously process more than one source of information. The null effects of optic flow may also be the result of the high friction Dycem material that covered the treadmill belt. Further work is clearly warranted to understand how combinations of sensory input influence early stepping and how early locomotor interventions can be optimized. To clarify why optic flow did not have an effect on forward stepping in the current experiment it will be particularly important to compare stepping rates when newborns are exposed to optic flow alone, a moving treadmill alone (with and without Dycem), and a combination of optic flow and a moving treadmill. If future studies confirm that Dycem facilitates newborn stepping, this will be an important finding in its own right because it will suggest a simple way to enhance the effectiveness of early treadmill stepping interventions.

## Data Availability Statement

The original data presented in the study are included in the article, further inquiries can be directed to the corresponding author.

## Ethics Statement

The studies involving human participants were reviewed and approved by the Comité ethique Ile de France 3. Written informed consent to participate in this study was provided by the participants’ legal guardian/next of kin.

## Author Contributions

MB-R: design of the study, ethical approval, supervision of the experiment, and writing. KS: design of the study, ethical approval of the study, participant testing, analysis, and writing. DA: design of the study and writing. VH: testing and analysis. AD: analysis. FG: supervision of the study. CT: design of the study, testing, analysis, and writing. All authors contributed to the article and approved the submitted version.

## Conflict of Interest

The authors declare that the research was conducted in the absence of any commercial or financial relationships that could be construed as a potential conflict of interest.
